# Progesterone Promotes Mitochondrial Respiration at the Biochemical and Molecular Level in Germinating Maize Seeds

**DOI:** 10.3390/plants10071326

**Published:** 2021-06-29

**Authors:** Hulya Turk

**Affiliations:** East Anatolian High Technology Application and Research Center, Ataturk University, Erzurum 25240, Turkey; hulyaa.turk@hotmail.com; Tel.: +90-442-231-7268

**Keywords:** progesterone, mitochondrial respiration, gene expression, germination, maize

## Abstract

This research aimed to investigate the effects of progesterone, a mammalian steroid sex hormone, on the mitochondrial respiration in germinating maize seeds. For this purpose, maize seeds were divided into four different groups (control, 10^−6^, 10^−8^, and 10^−10^ mol·L^−1^ progesterone) and were grown in a germination cabinet in the dark at 24.5 ± 0.5 °C for 4 d. The changes in gene expression levels of citrate synthase (*CS*), cytochrome oxidase (*COX19*), pyruvate dehydrogenase (*Pdh1*), and ATP synthase (*ATP6*), which is involved in mitochondrial respiration, were studied in root and cotyledon tissues. Significant increases were recorded in the gene expression levels of all studied enzymes. In addition, progesterone applications stimulated activities of malate synthase (MS), isocitrate lyase (ICL), and alpha-amylase, which are important enzymes of the germination step. The changes in gene expression levels of *mas1* and *icl1* were found parallel to the rise in these enzymes’ activities. It was determined similar increases in root and coleoptile lengths and total soluble protein and total carbohydrate contents. The most remarkable changes were detected in 10^−8^ mol·L^−1^ progesterone-treated seedlings. These results clearly indicate that progesterone stimulates mitochondrial respiration by inducing biochemical and molecular parameters and thus accelerates seed germination thanks to the activation of other pathways related to mitochondrial respiration.

## 1. Introduction

Mammalian sex hormones (MSHs) are a member of steroids and are naturally present in plants [1]. After the determination of MSHs in plants, many studies were conducted regarding the presence, quantities, types, effects, and receptors of these steroids in various organs and tissues of many plant species in all developmental stages from germination to flowering [2,3,4,5]. The studies were focused on determining the effects of exogenous application of these steroids on the growth and development of plants, their tolerance to various stressors, and their mode of action in metabolism in recent years [6,7,8,9,10]. Among the MSHs, the most studied hormone concerning its effects on plants is progesterone. The presence of progesterone in plants was first reported by Gawienowski and Gibbs in the 1960s [11]. Like other MSHs, progesterone (PRG) also has a generally stimulating effect on all developmental stages of plants from germination to the flowering stage, and this effect was clearly revealed by the findings obtained from the studies regarding its effects on many parameters such as cell division, callus formation, root-stem elongation, pollen germination, protein, sugar, pigment, and phenolic substance contents in various plants [1,12,13,14]. Moreover, progesterone was shown to have an improving effect on plant defense system activation against various biotic-abiotic environmental stress factors by activating both enzymatic and non-enzymatic antioxidant defense systems, and thus oxidative stress parameters [3,9,15,16,17]. In spite of all these promoting effects of progesterone on plant growth and development and defense system, its mode of action is still not completely clarified. Many studies are still being conducted on whether its effects occur directly by itself or indirectly by affecting different metabolic pathways or components. Recently, studies on progesterone biosynthesis inhibitors and receptor binding inhibitors gained momentum in order to elucidate its mechanism of action. Janeczko et al. [3] reported that mifepristone, a receptor-binding inhibitor of progesterone, with and without progesterone, led to severe growth attenuation in wheat seedlings. In mammals, mitochondria have specific progesterone receptors [18]. When bound to receptors, progesterone increases the cellular respiration level by raising the mitochondrial membrane potential [18]. In a previous study, Erdal and Genisel [17] reported that this steroid modulates the rates of anabolic and catabolic reactions in cells by affecting mitochondrial respiration. In the literature, to our best knowledge, there is no investigation reporting the effect of progesterone on mitochondrial respiration, the crossroads of many metabolic processes, in germinating seeds.

In the present study, we aimed to enlighten based on physiological, biochemical, and molecular features the possible stimulating effect of progesterone on seed germination and early growth parameters by considering the importance of mitochondrial respiration in the stage of seed germination. This is the first detailed investigation-exhibiting role of progesterone on mitochondrial respiration in the stage of seed germination and early plant growth.

## 2. Results

### 2.1. Seeds Germination Rate and Root and Coleoptile Length

**Figure 1:**
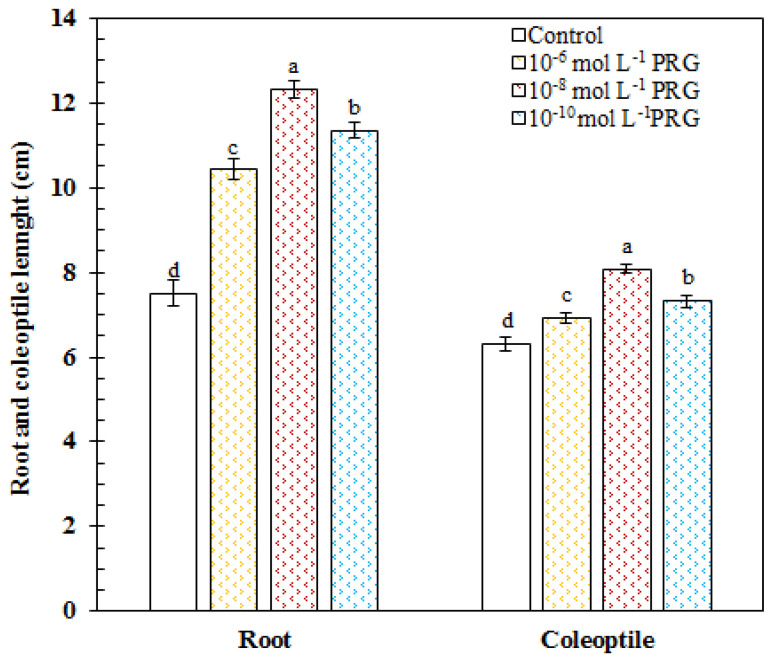
Effects of progesterone applications on root and coleoptile lengths of 4-day-old maize seedlings.

In the control group, the root and coleoptile length was measured as 7.51 and 6.32 cm, respectively. In 10^−6^, 10^−8^, and 10^−10^ mol·L^−1^, progesterone supplementations markedly increased the root length by 38.79, 63.96, and 51.17%, respectively, as compared to the control group. The values of the root length were recorded as 10.42, 12.31, and 11.35 cm in progesterone-treated seeds. In 10^−6^, 10^−8^, and 10^−10^ mol·L^−1^, progesterone supplementations significantly enhanced the coleoptile length by 9.37, 27.89, and 15.86%, respectively, as compared to the control group. The values of the coleoptile length were recorded as 6.91, 8.08, and 7.32 cm in progesterone-treated seeds (Figure 1).

### 2.2. Total Soluble Protein Content and Total Carbohydrate Content

Total soluble protein and carbohydrate contents were determined in the root, coleoptile, and endosperm tissues. In roots, progesterone applications increased by 30.03, 39.87, and 35.09% of protein contents in comparison to the control, respectively. The values of the protein content were recorded as 13.82, 17.97, 19.33, and 18.67 mg·g^−1^ FW in the control group, and 10^−6^, 10^−8^, and 10^−10^ mol·L^−1^ progesterone applications, respectively. In coleoptiles, progesterone applications increased by 31.08, 47.60, and 32.16% of protein contents in comparison to the control, respectively. The values of the protein content were recorded as 18.53, 24.29, 27.5, and 24.49 mg·g^−1^ FW in the control, and 10^−6^, 10^−8^, and 10^−10^ mol·L^−1^ progesterone applications, respectively. In endosperm tissues, progesterone applications increased by 18.77, 29.22, and 20.11% protein contents in comparison to the control, respectively. The values of the protein content were recorded as 12.73, 15.12, 16.45, and 15.29 mg·g^−1^ FW in the control, 10^−6^, 10^−8^, and 10^−10^ mol·L^−1^ progesterone applications, respectively (Table 1).

In roots, progesterone applications increased by 22.62, 33.91, and 27.13% the content of total carbohydrate in comparison to control, respectively. The values of the total carbohydrate content were recorded as 31.52, 38.65, 42.21, and 40.07 mg·g^−1^ FW in the control, 10^−6^, 10^−8^, and 10^−10^ mol·L^−1^ progesterone applications, respectively. In coleoptiles, progesterone applications increased by 17.45, 26.71, and 22.60% total carbohydrate contents in comparison to the control, respectively. The values of the total carbohydrate content were recorded as 35.31, 41.47, 44.74, and 43.29 mg·g^−1^ FW in the control and 10^−6^, 10^−8^, and 10^−10^ mol·L^−1^ progesterone applications, respectively. In endosperm tissues, progesterone applications decreased by 15.34, 21.94, and 12.14% total carbohydrate contents in comparison to the control, respectively. The values of the total carbohydrate content were recorded as 133.34, 112.88, 104.08, and 117.15 mg·g^−1^ FW in the control, and 10^−6^, 10^−8^, and 10^−10^ mol·L^−1^ progesterone applications, respectively (Table 1).

### 2.3. Enzymes Activities Related to Germination Phase

The activities of amylase, isocitrate lyase, and malate synthase were presented in Table 2. Progesterone applications (10^−6^, 10^−8^, and 10^−10^ mol·L^−1^) exhibited significant increases in endosperms of maize seedlings compared to the control group of all of the enzymes studied.

The amylase activity increased by 12.58, 27.65, and 14.92% in 10^−6^, 10^−8^, and 10^−10^ mol·L^−1^ progesterone in comparison to the control, respectively. The values of the amylase activity were recorded as 73.19, 82.40, 93.43, and 84.11 U mg protein^−1^ FW in the control, and 10^−6^, 10^−8^, and 10^−10^ mol·L^−1^ progesterone applications, respectively.

As seen in Table 2, compared to the control, isocitrate lyase activity enhanced by 34.33, 67.10, and 30.81% in 10^−6^, 10^−8^, and 10^−10^ mol·L^−1^ progesterone applications, respectively. The values of the ICL activity were recorded as 1.53, 2.06, 2.56, and 2.00 U mg protein^−1^ FW in the control, and 10^−6^, 10^−8^, and 10^−10^ mol·L^−1^ progesterone applications, respectively.

Malate synthase activity caused remarkable increases by 43.70, 90.44, and 49.80% in 10^−6^, 10^−8^, and 10^−10^ mol·L^−1^ progesterone compared with the control group, respectively. The values of the MS activity were recorded as 7.17, 10.31, 13.66, and 10.74 U mg protein^−1^ FW in the control, and 10^−6^, 10^−8^, and 10^−10^ mol·L^−1^ progesterone applications, respectively.

### 2.4. Expression of Isocitrate Lyase, Malate Synthase, Citrate Synthase, Cytochrome Oxidase, Pyruvate Dehydrogenase, and ATP Synthase

**Figure 2:**
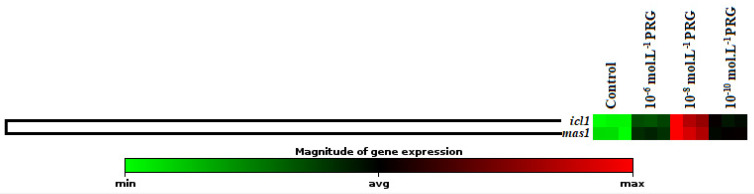
Effect of progesterone on the gene expression of isocitrate lyase (*icl1*) and malate synthase (*mas1*) in the endosperms of 4-day-old maize seedlings. The clustergram performs non-supervised hierarchical clustering of the entire dataset to display a heat map with dendrograms indicating co-regulated genes across groups.

**Figure 3:**
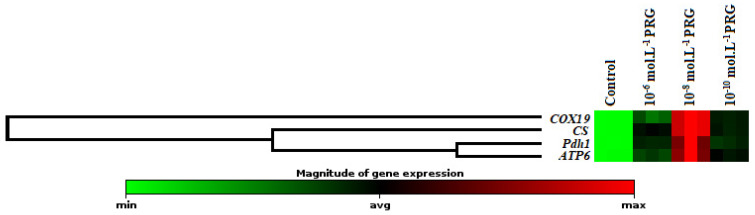
Effect of progesterone on the gene expression of citrate synthase (*CS*), cytochrome oxidase (*COX19*), pyruvate dehydrogenase (*Pdh1*), and ATP synthase (*ATP6*) in the coleoptiles of 4-day-old maize seedlings. The clustergram performs non-supervised hierarchical clustering of the entire dataset to display a heat map with dendrograms indicating co-regulated genes across groups.

**Figure 4:**
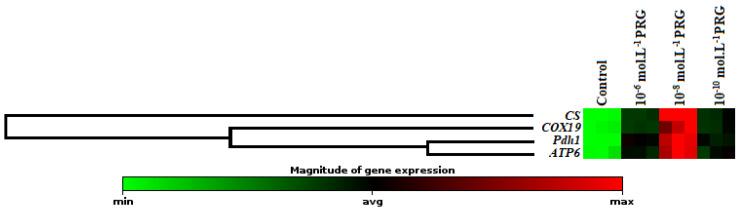
Effect of progesterone on the gene expression of citrate synthase (*CS*), cytochrome oxidase (*COX19*), pyruvate dehydrogenase (*Pdh1*), and ATP synthase (*ATP6*) in the roots of 4-day-old maize seedlings. The clustergram performs non-supervised hierarchical clustering of the entire dataset to display a heat map with dendrograms indicating co-regulated genes across groups.

The gene expression levels of these enzymes were also investigated to clarify the effects of progesterone application on maize seeds (Figure 2, Figure 3 and Figure 4). In addition to enzyme activities, *icl1* and *mas1* gene levels increased under the 10^−6^, 10^−8^, and 10^−10^ mol·L^−1^ progesterone supplementation compared to the control (Figure 2). Furthermore, *CS, COX19, Pdh1,* and *ATP6* gene levels were upregulated by progesterone supplementations compared with the control in both root and coleoptile (Figure 3 and Figure 4). 

## 3. Discussion

The present investigation aimed, for the first time, to reveal the effects of exogenous progesterone application on early seedling growth based on the changes in particularly mitochondrial respiration besides physiological ad biochemical parameters in germinating maize seeds. All applied concentrations of progesterone significantly increased the root and coleoptile lengths of maize seedlings in comparison to their controls. The best ameliorative effect on these parameters was obtained at 10^−8^ mol·L^−1^ progesterone-applied seedlings (Figure 1). Similar to our findings, in research carried on 7-day-old maize seedlings, 10^−8^ mol·L^−1^ progesterone application exhibited the most stimulating effect on the lengths of root and seedling under salt stress in wheat [6]. In another study, it was reported that exogenous progesterone applications increased root and shoot lengths in winter wheat [19]. External differentiation in plants is shaped by changes in the types and levels of proteins and carbohydrates found in internal tissues [20]. For this reason, changes in soluble protein and carbohydrate content are considered indicators of growth and development [21]. Especially, decreases in protein content affect plant growth negatively. We found that total soluble protein content was higher than those of control plants in the root, coleoptile, and endosperm tissues of progesterone-applied seedlings (Table 1). Consistent with our findings, Genisel et al. [22] reported that progesterone application resulted in a stimulative effect on the protein content of 16-day-old chickpea seedlings exposed to chilling stress. Erdal [6,7] informed that exogenous progesterone application increased soluble protein content in germinating maize seeds and wheat seedlings exposed to salt stress. On the other side, seeds accumulate the storage materials such as carbohydrates in endosperm or cotyledons [23] for supplying the required energy during germination. While progesterone applications increased total carbohydrate contents in root and coleoptile, it led to a marked decrease in endosperm tissue in comparison to their controls (Table 1). In a previous study, Erdal and Dumlupinar [24] informed that progesterone application enhanced total carbohydrate amount in germinating chickpea seeds. These findings put forward that progesterone has a significant role on early seedling growth due to its enhancing effect on protein and carbohydrate contents.

In the beginning stage of germination, the production of hydrolyzing enzymes such as alpha-amylase is significantly induced [24]. Alpha-amylase is a critical enzyme in seed germination and has a key role in the degradation of stored carbohydrates to soluble sugars used for supplying the required energy in the germination stage [25,26]. In this investigation, the alpha-amylase activity was increased by all the applied progesterone concentrations in respect to that of control seedlings. It is highly likely that a decrease in total carbohydrate content resulted from progesterone-induced elevation in alpha-amylase activity in endosperm tissues. As shown in Table 2, 10^−8^ mol·L^−1^ progesterone treatment exhibited the highest activity values in alpha-amylase activity. This finding is in accordance with the results of previous researchers, who showed that progesterone stimulated the alpha-amylase activity in germinating seeds [13,21,24].

Isocitrate lyase (ICL) and malate synthase (MS) have major effects in the mobilization of storage lipids during germination [27,28]. These enzymes participate in the reactions of the glyoxylate cycle, which provides the conversion of two molecules of acetyl coenzyme-A to succinate for carbohydrates [29]. The intermediate metabolic product is, at last, converted into saccharose to serve as a primary nutrient source used to grow until the beginning of the photosynthetic activity in seedlings. While ICL catalyzes the conversion of isocitrate to succinate and glyoxylate molecule, MS catalyzes the conversion of glyoxylate to malate. In the present investigation, we found that progesterone application, especially 10^−8^ mol·L^−1^ progesterone, increased ICL and MS activities in maize endosperm tissues compared to the control (Table 2). There is no study showing the effects of progesterone on ICL and MS activity in the literature. In addition to ICL and MS enzyme activities, progesterone applications remarkably augmented the gene expression levels of these enzymes (Figure 2). The obtained findings in alpha-amylase, ICL, and MS activities and gene expression levels showed a significant agreement with the progesterone-induced increments in growth parameters, including coleoptile and root length, total soluble protein contents, and total carbohydrate content.

In the present investigation, the changes in gene expression levels of citrate synthase (*CS*), cytochrome oxidase (*COX19*), pyruvate dehydrogenase (*Pdh1*), and ATP synthase (*ATP6*) were also analyzed to enlighten the effect of progesterone on the mitochondrial respiration pathway. The expression levels of genes encoding *CS* and *COX19* were presented in Figure 3 and Figure 4. While CS is known as the first enzyme of the Krebs cycle, COX is the membrane-bound terminal enzyme in the electron transfer chain. Gene expression levels of these enzymes were significantly upregulated by progesterone applications in both root and coleoptile tissues in comparison to their control groups. Progesterone induction enhanced the expressions of CS, and COX19 proteins revealed that progesterone contributed to the acceleration of plant growth thanks to the formation of the extra energy needed as a result of stimulation of tricarboxylic acid cycle (TCA) and electron transport chain (ETS). Pyruvate dehydrogenase is the first component enzyme of the pyruvate dehydrogenase multienzyme complex (PDC). This complex catalyzes the conversion of pyruvate into acetyl-CoA units, thus binding glycolysis with the Krebs cycle. Progesterone applications significantly upregulated the gene expression of *Pdh1* compared to control in both root and coleoptile tissues of maize (Figure 3 and Figure 4). Similar to the *Pdh1* gene expression level, progesterone showed the same effects on the *ATP6* gene expression, as well (Figure 3 and Figure 4). ATP synthase, located in the ETS chain, directly produces ATP, a major energy compound used in cells, throughout the operation of cellular respiration [30,31]. These data put forward to that progesterone has a regulatory effect on mitochondrial respiration to supply high requirement of ATP, needed for maintaining seed germination and plant growth. Besides, the hierarchical cluster with a heap map clearly demonstrated the co-regulating effect of progesterone on the gene expressions of mitochondrial respiration-related enzymes (Figure 3 and Figure 4). There is limited study exhibiting the effect of progesterone on the mitochondrial respiration pathway in the literature. Erdal and Genisel [17] reported that progesterone application enhanced the total respiration rate compared with the control seedlings under normal and cold conditions in maize. In this study, respiratory rate (Vt), cytochrome respiratory capacity (Vcyt), alternative respiratory capacity (Valt), Valt/Vt, and Vcyt/Vt were determined from 14-day-old maize seedlings. The recorded increases in the mitochondrial respiration pathway may be an important indicator of the activation of the other stages of respiration, including the Krebs cycle. When these findings are taken together, it is possible to say that the respiratory-sourced raw materials are sufficiently formed and so the plants growth and development may be stimulated under normal and stressed conditions.

In conclusion, the present study clearly showed that progesterone applications stimulated mitochondrial respiration in both root and coleoptile tissues of maize at the level of biochemical and molecular. Furthermore, progesterone stimulated alpha-amylase, isocitrate lyase, and malate synthase, important for the germination stage, at the level of activity and gene expression. As expected, progesterone also performed a stimulating effect on root and coleoptile lengths and total soluble protein and total carbohydrate contents, and thus resulted in a remarkable increase in seedling growth. On the other hand, determining the most effective concentration of progesterone as 10^−8^ mol·L^−1^ is very important for its future usability due to the minimization of its possible reverse effects for human and animal health. However, further studies are needed on various crop species to prove the usability and credibility of this steroid in agricultural applications.

## 4. Materials and Methods

### 4.1. Seed Germination Conditions and Progesterone Treatments

Maize seeds (*Zea mays* L. cv. Hido) were used for this experiment. Firstly, all seeds were subjected to surface sterilization by using 96% ethanol for a short time, and then 5% NaClO for 10 min and washed with ultra-distilled water several times. After sterilization, the seeds were divided into four different groups; control group, 10^−6^ mol·L^−1^ PRG, 10^−8^ mol·L^−1^ PRG, and 10^−10^ mol·L^−1^ PRG. The optimum concentrations obtained in the present study were determined according to the previous studies in the literature. The concentration range was chosen from 10^−4^ to 10^−12^ mol·L^−1^ progesterone. After the pre-experiments, this range was determined as 10^−6^, 10^−8^, and 10^−10^ mol·L^−1^ progesterone. To prepare the stock solution (10^−4^ mol·L^−1^), progesterone was weighed and dissolved first in a small volume of methanol (2 or 3 mL for 1 L solutions), and the volume of solution was completed with sterile ultra-distilled water. Then the prepared stock solution was diluted in sterile ultra-distilled water in order to obtain 10^−6^, 10^−8^, and 10^−10^ mol·L^−1^ concentrations. The sterilized seeds were shared equally among all groups and were imbibed in their own solutions. After the imbibition period, ten seeds per dish were placed in Whatman No. 1 filter paper-lined glass Petri dishes. Each treatment group was supplied with its own progesterone solutions, while control seeds were supplied with sterile ultra-distilled water containing methanol (2 or 3 mL for 1 L). All groups were incubated in a germination cabinet, which was set at 24.5 ± 0.5 °C, and under dark conditions for 4 d. On the 4th day, all seedlings were harvested, and endosperm, coleoptile, and roots of seedlings were separated and stored at −86 °C until the determination of changes in physiological, biochemical, and molecular parameters.

### 4.2. Determination of Root and Coleoptile Lengths

After the harvest, the root and the coleoptile lengths of maize seedlings were measured by a centimetric ruler.

### 4.3. Determination of Total Soluble Protein and Carbohydrate Contents

The amount of total soluble protein was estimated according to Smith et al. [32] by using the bicinchoninic procedure. The 0.5 g tissue samples were homogenized with 100 mM KH_2_PO_4_ buffer (pH 6.75), and samples were centrifuged at 15,000 rpm at 4 °C for 15 min. After the centrifugation period, 5 μL supernatant was transferred to wells of the plate, and BCA reagent (bicinchoninic acid + CuSO_4_) was added into the samples. The plate was incubated at 65 °C for 15 min. The changes of absorbance were recorded at 562 nm, and protein values were determined by utilizing the standard chart.

The total carbohydrate content was determined according to the method described by Dische [33] with a slight modification. Tissue samples (100 mg) were homogenized with 5 mL of 2.5 N HCl. The homogenates were incubated for 1 h at 100 °C. After the cooling period, Na_2_CO_3_ powder was added into samples until the reaction stopped. An amount of 45 mL of distilled water was added on top of the samples, and they were centrifuged at 15,000 rpm for 15 min. After the centrifugation, 1 mL of supernatant was taken, and an anthrone solution was added. After cooling, the absorbance changes were recorded at 630 nm. The standard chart was used to determine the total carbohydrate content.

### 4.4. Determination of the Enzyme Activities Related to the Germination Phase

Amylase activity was determined according to the procedure defined by Juliano and Varner [34]. The endosperm tissues were homogenized with 50 mM (pH 7) tris-maleate buffer, and samples were centrifuged at 2400× *g* for 20 min. After the centrifugation, 500 μL of supernatant was mixed with 500 μL 40 mM EDTA and was added into 1 mL of reaction buffer including 200 μmol CaCl_2_, 150 mg of soluble starch, and 600 mg of KH_2_PO_4_ dissolved in 100 mL of distilled water. The samples were incubated at 30 °C for 5 min. Then, the reaction was stopped by adding 1 mL of iodine reagent (3 mg of KI and 0.3 mg of I were dissolved in 0.05 N HCl). Thirteen milliliters of distilled water were added to the samples, and absorbance changes were recorded at 620 nm. One unit of amylase activity was identified as the quantity of enzyme that caused the changes in the absorbance of 0.1 [35].

For the extraction of isocitrate lyase and malate synthase activity, 10 g of endosperm tissue was extracted in extraction buffer containing 400 mM sucrose, 165 mM N-trishydroxymethy glycine (pH 7.5), and 10 mM dithiothreitol. The samples were centrifuged at 270× *g* for 10 min. After centrifugation, the pellet was taken and was homogenized 6 mL of extraction buffer. All samples were homogenized at 23,000 rpm for 5 h.

Isocitrate lyase activity was conducted by the method of Dixon and Kornberg [36]. Enzyme activity was quantified using the molar extinction coefficient for (1.7 × 104 cm^−1^ M^−1^). About 15 μg of supernatant was mixed with 1.2 mL of reaction buffer, including 87 mM phosphate buffer (pH 6.9), 4.6 mM dithiothreitol, 8.7 mM MgCl_2_, 13 mM isocitrate, and 10 mM phenylhydrazine. The reaction was started by adding the isocitrate to the mixture, and the absorbance values were determined at 324 nm.

Malate synthase activity was determined according to Hock and Beevers [37]. About 25 μg of supernatant was added into reaction buffer containing 77 mM tris (pH 8), 1.5 mM dithiobis(nitrobenzoic acid), 7.7 mM MgCl_2_, 18 mM acetyl coenzyme-A, and 20 mM sodium glyoxylate. The values of absorbance were recorded at 412 nm.

### 4.5. The Isolation of Total RNA and RT-PCR Analysis

The isolation of total RNA and quantitative real-time PCR reactions were performed according to the producer’s analysis protocol. All tissue samples were conducted in a Qiacube device by using RNeasy plant mini kit (Qiagen, Hilden, Germany) for RNA isolation. The RNA’s purity of samples was measured at the Qiaexpert. cDNA synthesis was conducted by producer’s procedures by using Nanoscript 2 RT kit (Primer Design). Specific gene primers were provided from Qiagen Company. The relative gene expression analysis was conducted by Qiagen-Geneglobe [38]. β-actin was preferred as a housekeeping gene. The experiment was randomly conducted with three independent biological replications and three parallel. The final results of *icl1, mas1, CS, COX19, Pdh1*, and *ATP6* mRNA accumulation were given as a heat map with dendrograms indicating co-regulated genes. The primer series of the genes were presented in Table 3.

### 4.6. Statistical Analysis

The assay was randomly conducted with five biological replications and three parallel. To compare significant differences between the samples was used the analysis of variance (ANOVA). Statistical significance was defined as *p* < 0.05 (Duncan’s multiple range method). The statistical analyses were conducted using SPSS 20.0, and the standard errors were shown in all tables and figures.

## Figures and Tables

**Table 1:** Effects of progesterone applications on soluble total protein content and total carbohydrate content in root, coleoptile, and endosperm tissues in 4-day-old maize seeds.

Treatments	Soluble Protein Content (mg·g^−1^ FW)			Total Carbohydrate Content (mg·g^−1^ FW)		
	Root	Coleoptile	Endosperm	Root	Coleoptile	Endosperm
Control	13.82 ^c^	18.52 ^c^	12.73 ^c^	31.52 ^d^	35.31 ^d^	133.34 ^a^
10^−6^ mol·L^−1^ PRG	17.97 ^b^	24.29 ^b^	15.12 ^b^	38.65 ^c^	41.47 ^c^	112.88 ^b^
10^−8^ mol·L^−1^ PRG	19.33 ^a^	27.35 ^a^	16.45 ^a^	42.21 ^a^	44.74 ^a^	104.08 ^c^
10^−10^ mol·L^−1^ PRG	18.67 ^ab^	24.49 ^b^	15.29 ^b^	40.07 ^b^	43.29 ^b^	117.15 ^b^

Different letters in the same group indicate statistically significant differences (*p* < 0.05).

**Table 2:** Effects of progesterone applications on α-amylase, isocitrate lyase, and malate synthase activities in 4-day-old maize endosperms.

Treatments	α-Amylase (U mg Protein^−1^ FW)	Isocitrate Lyase (U mg Protein^−1^ FW)	Malate Synthase (U mg Protein^−1^ FW)
Control	73.19 ^c^	1.53 ^c^	7.17 ^c^
10^−6^ mol·L^−1^ PRG	82.40 ^b^	2.00 ^b^	10.31 ^b^
10^−8^ mol·L^−1^ PRG	93.43 ^a^	2.56 ^a^	13.66 ^a^
10^−10^ mol·L^−1^ PRG	84.11 ^b^	2.06 ^b^	10.74 ^b^

Different letters in the same group indicate statistically significant differences (*p* < 0.05).

**Table 3:** Primer sequences of the genes studied for RT-PCR analysis.

Enzyme’s Name	Target Gene	Primer Sequences
*Isocitrate lyase*	*icl1* forward	5′-GAGATGGCCAAGAAGCTGTG-3′
	*icl1 reverse*	5′-GTAGATGGTGTCCAGGTGCT-3′
*Malate synthase*	*mas1* forward	5′-TCGACTTCGGCCTCTACTTC-3′
	*mas1 reverse*	5′-ATCCTCGCTTCTCTGGAGTG-3′
*Citrate synthase*	*CS* forward	5′-TGCTCACAGTGGAGTTTTGC-3′
	*CS reverse*	5′-AACACTCTTCGGCCTCTCAA-3′
*Cytochrome oxidase*	*COX19* forward	5′-CATGAGTGCGACTTGGAGAA-3′
	*COX19 reverse*	5′-TCAGGAGATGTACCCGCTTC-3′
*Pyruvate dehydrogenase*	*Pdh1* forward	5′-CTCAACATTTCGGCCCTCTG-3′
	*Pdh1 reverse*	5′-CATAGTCGCCACGCTTGTAG-3′
*ATP synthase*	*ATP6* forward	5′-CACTTAACGAGCACCACCAG-3′
	*ATP6 reverse*	5′-GGATCCTGCAGACTCTCTCC-3′
*β*-actin	*actb1* forward	5′GTGACAATGGCACTGGAATG-3′
	*actb1* reverse	5′-CCATGCTCAATCGGGTACTT-3′

## Data Availability

Not applicable.
